# Impacts of salt restriction on nutritional status, sarcopenia, and mortality of cirrhotic patients with ascites

**DOI:** 10.1186/s12876-025-03830-1

**Published:** 2025-07-15

**Authors:** Maha Elsabaawy, Mohammed Ragab, Madiha Naguib, Eman Kamal, Maymona Al-Khalifa, Khaled Gamil, Marwa Elfayoumy

**Affiliations:** 1 Hepatology and Gastroenterology Department, National Liver Institute Minufiya University, Shebeen Elkoom, Egypt; 2 Anesthesia and Intensive Care Nutrition Department, National Liver Institute Menoufia University, Shebeen Elkoom, Egypt; 3 Therapeutic Nutrition Department, National Liver Institute Menoufia University, Shebeen Elkoom, Egypt

**Keywords:** Cirrhosis, Ascites, Salt restriction, Nutritional status, Malnutrition, Sodium intake

## Abstract

**Background:**

Salt restriction is a cornerstone in managing ascites in cirrhotic patients; however, its impact on nutritional status, sarcopenia, and mortality remains unclear.

**Aim:**

To evaluate the effects of a salt-restricted diet (SRD) on ascites control, body composition, sarcopenia development, and patient survival in cirrhotic patients with decompensated liver disease.

**Methods:**

This prospective study included 102 patients with grade III ascites, categorized into two groups based on dietary adherence: Salt-Restricted Diet (SRD), (*n* = 46) and Salt-Unrestricted Diet (SUD) (*n* = 56). Sodium intake was assessed using the Dietary Sodium Restriction Questionnaire (DSRQ) and spot urine Na/K ratio. Nutritional status, sarcopenia, ascites control, and six-month mortality were evaluated.

**Results:**

The SRD group showed better ascites control, with fewer paracentesis sessions per month (1.57 ± 0.65 vs. 4.07 ± 1.43, *p* < 0.001). Sarcopenia was more prevalent in the SRD group (*p* < 0.001), with lower SMI (4.88 ± 7.13 vs. 16.7 ± 544.8, *p* < 0.001) and TR PMM (19.03 ± 3.68 vs. 71.92 ± 191.9, *p* < 0.001). Higher nutritional risk was significantly associated with SRD (*p* = 0.001). Mortality was significantly higher in the SRD group (67.4%) compared to the SUD group (35.7%), *p* = 0.001. Multivariate analysis identified sarcopenia (OR = 2.684, *p* = 0.006) and SRD (OR = 1.65, *p* < 0.001) as independent predictors of mortality.

**Conclusion:**

While effective in ascites control, sodium restriction may compromise nutritional status, heighten sarcopenia risk, and increase mortality, highlighting the need for a more individualized dietary approach.

## Introduction

Cirrhosis, a chronic liver condition characterized by irreversible fibrosis, poses significant challenges in clinical management, particularly in advanced stages when complications such as ascites develop [1]. Approximately 50% of cirrhotic patients develop ascites within ten years of diagnosis, making management a central component of care for this population [2]. Sodium retention plays a pivotal role; therefore, dietary sodium restriction is widely recommended as a first-line intervention to manage ascites [3–5]. Cirrhotic patients with ascites are advised to limit their salt intake, with recommendations ranging from 4.6 to 6.9 g daily, with most guidelines recommending around 5 g (equal to a teaspoon of table salt) [3–5].

However, despite its long-standing use, the role of strict salt restriction in ascites management remains a subject of debate. While sodium restriction aims to control fluid retention, emerging evidence suggests that excessive restriction may negatively impact nutritional status, muscle mass, and overall prognosis. Cirrhotic patients are already at high risk of protein-energy malnutrition and sarcopenia due to factors such as anorexia, hypermetabolism, and impaired nutrient absorption. Severe sodium restriction may exacerbate these issues, leading to increased catabolism, muscle wasting, and worse clinical outcomes [6–11].

Despite the widespread adoption of sodium restriction, there is a lack of longitudinal studies systematically evaluating both its benefits and potential risks, particularly regarding its impact on sarcopenia, nutritional status, and mortality. Previous studies have primarily focused on short-term ascites control, overlooking the long-term consequences of dietary sodium restriction on body composition, functional status, and survival. Furthermore, the relationship between sodium intake, paracentesis frequency, and nutritional deterioration remains poorly understood.

Given this knowledge gap, our study aims to assess the impact of salt restriction on sarcopenia, nutritional status, and mortality in cirrhotic patients with ascites. By evaluating these parameters over a six-month follow-up period, we seek to provide a more comprehensive understanding of the risks and benefits of sodium restriction and challenge the long-held assumption that strict sodium limitation is universally beneficial in this patient population. Accordingly, this study was designed to evaluate the impact of salt restriction on nutritional status, muscle mass, mortality added to ascites control in patients with liver cirrhosis.

## Patients and methods

This observational prospective cohort study included 115 patients diagnosed with decompensated liver cirrhosis with grade III ascites at the Paracentesis Unit in the Hepatology and Gastroenterology Department at the National Liver Institute (NLI), Menoufia University, from 15 th March 2023 to 23rd September 2024.

The study was conducted upon approval of the Ethical Committee Board of NLI and informed consent to participate was obtained from all the participants in the study.

Included cases were patients of both sexes, over 18 years old, diagnosed with cirrhosis by clinical (jaundice, ascites, variceal bleeding, or encephalopathy) and by ultrasound liver surface nodularity and portal vein mean flow velocity [12], and Patients with grade 3 (large or gross ascites, marked distension of the abdomen, refractory ascites, ascites that cannot be mobilized, or early recurrence (i.e., after large volume paracentesis (LVP)) cannot be satisfactorily prevented by medical therapy [13].

Exclusion criteria were patients with comorbidities that can mimic cirrhotic ascites such as heart and lung insufficiency and patients with previous renal impairment; patients diagnosed with hepatocellular carcinoma or other malignancies; active drug or alcohol abuse; active complications of spontaneous bacterial peritonitis; hepatic encephalopathy; gastrointestinal bleeding within 2 weeks; hemorrhagic ascites, malignant ascites, ongoing hepatic encephalopathy, and/or another cognitive disorder that precludes the patient's ability to participate in the study; and other comorbidities whose management required specific dietary measures such as diabetes mellitus and chronic kidney disease.

### Group allocation

**Fig. 1:**
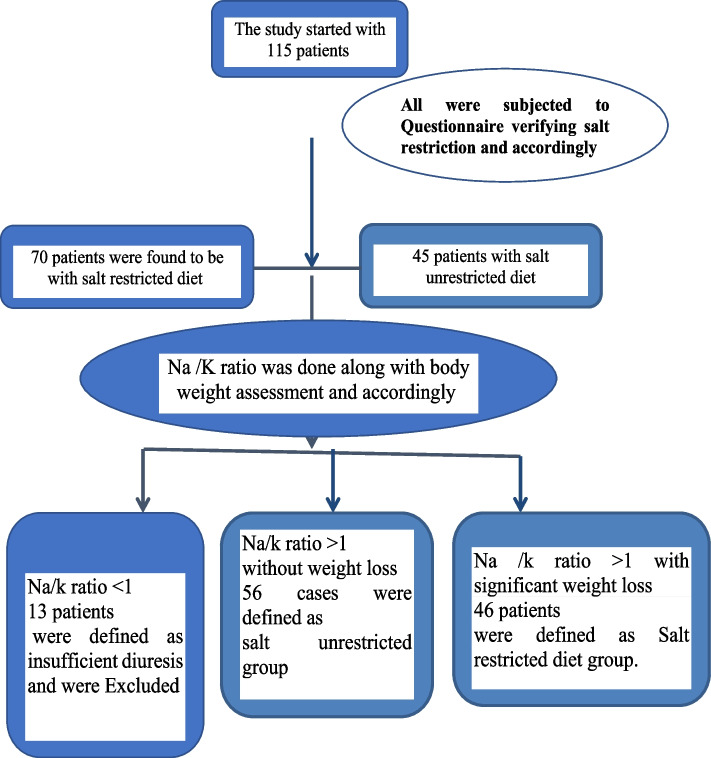
Flow chart of included cases

Patients were divided into two groups based on their dietary adherence: the salt-restricted diet (SRD) group and the salt-unrestricted diet (SUD) group. Allocation was performed according to the Dietary Sodium Restriction Questionnaire (DRSQ) questionnaire in two groups [14]. The urinary spot test along with weight loss assessment led to the exclusion of 13 cases with urinary Na/K and insignificant weight loss following both diuresis and abdominal paracentesis [15] (Fig. 1). The remaining 102 patients were grouped as follows: 46 with SRD and 56 with SUD.

Sarcopenia, and nutritional status were evaluated once at baseline to compare its prevalence between the two groups. Patients were then followed for six months to assess how salt restriction influenced mortality.

All patients were subjected to the collection of all baseline recruitment data: demographic data (age, sex, distribution, occupation, smoking, and alcohol intake), etiology of liver disease, time since the diagnosis of cirrhosis, time since the appearance of ascites, type of ascites (responsive or refractory) according to the criteria of the International Club of Ascites [16], and frequency of paracentesis. Laboratory investigations include prothrombin activity, index normalized ratio (INR), serum total bilirubin, albumin, creatinine, urea, and the serum concentration of sodium and potassium and a random “spot” urine sodium concentration and (K) concentration.

#### Questionnaire

All patients included in this study were interviewed by the same operator. The DSRQ, which were translated into the Arabic language. The DSRQ is an assessment instrument used to measure patients’ perceptions of their barriers to and attitudes toward following a low-sodium diet. The original instrument is divided into three subscales: 1) attitude; 2) subjective norms; and 3) perceived behavioural control [14]. The attitude subscale comprises six items and assesses patients’ beliefs about the results of adopting a given behaviour. The subjective norm subscale consists of three items and refers to the importance of the patient’s perception that others approve or disapprove of performing the behaviour. Finally, the perceived behavioural control subscale comprises seven items and evaluates the patient’s ability to identify facilitators and barriers related to behaviour.

The spot urine Na/K ratio was evaluated for the objective assessment of actual diuresis.

#### Nutritional assessment

Patients also underwent nutritional assessment using the Royal Free Hospital-Nutritional Prioritizing Tool (RFH-NPT) [17].

#### Sarcopenia assessment

Patients were evaluated for sarcopenia at baseline using a Body Composition Analyzer Device at the Nutrition Department at the National Liver Institute [18]. (InBody S10 (multi-frequency segmental BIA) was used, explicitly stating its advantages in cirrhotic patients with fluid retention. It allowed better differentiation between intracellular and extracellular water. Additionally, it provides segmental analysis, reducing the impact of ascites and lower limb edema on whole-body composition estimates.

Monitoring of Ascites Management: The frequency of paracentesis performed in the month prior to enrolment and during the study period was recorded for each patient.

### Follow-up

Patients were followed up for a period of six months or till death.

### Sample size calculation

Our sample size estimation was based on detecting differences in mortality, given its critical clinical relevance in cirrhotic patients with ascites. Using the Fleiss formula for comparing two proportions, we estimated that a minimum of 35 patients per group was required to detect a 31.7% difference in mortality (67.4% vs. 35.7%) with 80% power at a 5% significance level. Since our final sample included 46 patients in the SRD group and 56 in the SUD group, we exceeded this requirement, ensuring adequate power for mortality analysis. Additionally, we conducted a post hoc power analysis to assess the study’s ability to detect differences in sarcopenia and nutritional deficiencies. The analysis revealed:

• Mortality: 90.0% power (sufficient)
• Sarcopenia: 81.4% power (adequate)

Accordingly, the study had adequate power for mortality and sarcopenia.

### Statistical analysis

Data were fed to the computer and analysed using IBM SPSS software package version 20.0. (Armonk, NY: IBM Corp) Qualitative data were described using numbers and percentages. The Kolmogorov–Smirnov test was used to verify the normality of the distribution, and quantitative data were described using range (minimum and maximum), mean, standard deviation, median, and interquartile range (IQR). The significance of the results was determined at the 5% level. The chi-squared test was used for categorical variables to compare the different groups. Fisher’s Exact or Monte Carlo correction for chi-square when more than 20% of the cells had an expected count of < 5. The student’s t-test for normally distributed quantitative variables was used to compare the two groups. The Mann–Whitney test was used to compare abnormally distributed quantitative variables between the two groups.

## Results

### Baseline characteristics

No significant differences were observed between the SRD and SUD groups in terms of sex distribution, age, etiology of cirrhosis, Child–Pugh classification, ALBI score, MELD score, or MELD-Na score (*P* > 0.05) (Table 1).

**Table 1:** Comparison between the two studied groups

			**SRD** **(*n* = 46)**		**SUD** **(*n* = 56)**		**Test of significance**	**P**
	**No**	**%**	**No**	**%**	**No**	**%**		
**Sex**								
Male	63	61.8	24	38.1	39	61.9	χ^2^ = 3.263	0.071
Female	39	38.2	22	56.4	17	43.6		
**Age (years) M** ± SD	58.85 ± 10.29		58.37 ± 11.33		59.25 ± 9.43		t = 0.428	0.669
**Cause of Cirrhosis**								
Not known	19	18.6	10	52.6	9	47.4	χ^2^ = 0.535	0.464
HBV	6	5.9	1	16.7	5	83.3		
HCV	68	66.7	30	44.1	38	55.9	χ^2^ = 0.079	0.778
**Child Score**							χ^2^ = 0.067	0.796
B	48	47.1	21	43.8	27	56.3		
C	54	52.9	25	46.3	29	53.7		
**ALBI grade**							χ^2^ = 0.179	0.672
Grade 2	62	60.8	29	46.8	33	53.2		
Grade 3	40	39.2	17	42.5	23	57.5		
ALBI score M ± SD	1.52 ± 0.49		1.51 ± 0.56		1.53 ± 0.43		U = 1273.500	0.922
**MELD Score**							U = 1095.50	0.194
M ± SD	14.02 ± 5.18		14.57 ± 4.99		13.57 ± 5.33			
**MELD Score Na**							U = 1157.50	0.380
M ± SD	115.8 ± 48.82		112.5 ± 46.16		118.6 ± 51.15			

*SRD* salt restricted, *SUD* salt unrestricted group, *IQR* Inter quartile range, *SD* Standard deviation, *U* Mann Whitney test, *t* Student’st-test, *p* p value for comparing between the two studied groups, *BMI* Body Mass Index, *SMI* Skeletal Muscle Index, *PMM* Segmental Muscle Mass, *TR PMM* Trunk muscle mass, *SMI* Skeletal muscle index, *ASM* Appendicular Skeletal Muscle, *Ht* Height, *PhA* Phase Angle

### Ascites control

Patients in the SUD group required significantly more frequent paracentesis per month compared to the SRD group (4.07 ± 1.43 vs. 1.57 ± 0.65, *P* < 0.001).

### Body composition

Patients in the SUD group had significantly higher weight, modified weight, extracellular water (ECW), intracellular water (ICW), total body water (TBW), and segmental muscle mass (PMM) (*P* < 0.001). Lower skeletal muscle index (SMI) in the SRD group (*p* < 0.001) (Table 2).

**Table 2:** Comparison between the two groups according to control of ascites, sarcopenia via BIA, and body composition indices

	**Total (*n* = 102)**	**Result of lab**		**Test of Sig**	**P**
		**SRD** **(*n* = 46)**	**SUD** **(*n* = 56)**		
**Frequency of tapping in one month M** ± SD	2.94 ± 1.69	1.57 ± 0.65	4.07 ± 1.43	U = 44.500^*^	< 0.001^*^
Weight M ± SD	75.02 ± 15.29	69.70 ± 13.02	79.39 ± 15.74	t = 3.341^*^	0.001^*^
Modified weight M ± SD	60.02 ± 12.24	55.76 ± 10.42	63.52 ± 12.59	t = 3.341^*^	0.001^*^
BMI M ± SD	26.86 ± 5.24	25.99 ± 4.88	27.58 ± 5.46	t = 1.541	0.127
**Modified BMI M ± SD**	21.49 ± 4.19	20.79 ± 3.91	22.07 ± 4.37	t = 1.541	0.127
**ECW**					
Min. – Max	11.60–24.90	11.60–19.60	13.60–24.90	t = 7.182^*^	< 0.001^*^
Mean ± SD	16.81 ± 2.73	15.06 ± 1.88	18.24 ± 2.48		
**ICW**					
Min. – Max	12.90–37.20	12.90–26.30	14.40–37.20	t = 5.862^*^	< 0.001^*^
Mean ± SD	20.47 ± 4.92	17.74 ± 3.15	22.71 ± 4.99		
**Serum sodium**					
Min. – Max	115.6–145.0	124.0–144.0	115.6–145.0	U = 1135.0	0.302
Median (IQR)	135.0 (129.0–137.0)	136.0 (129.0–137.0)	133.0 (129.0–138.0)		
**Serum potassium**					
Min. – Max	2.80–10.20	2.80–10.20	2.90–5.0	U = 1220.50	0.649
Median (IQR)	3.80(3.50–4.20)	3.85(3.5–4.30)	3.80 (3.55–4.20)		
TBW M ± SD	37.39 ± 10.53	33.84 ± 6.86	40.31 ± 12.08	U = 551.00^*^	< 0.001^*^
FMI M ± SD	7.60 ± 3.34	7.40 ± 3.27	7.77 ± 3.42	U = 1221.00	0.652
BMR ± SD	118.5 ± 319.9	63.80 ± 10.50	163.5 ± 428.1	775.00^*^	0.001^*^
TR PMM M ± SD	48.07 ± 144.1	19.03 ± 3.68	71.92 ± 191.9	235.500^*^	< 0.001^*^
SMI M ± SD	11.4 ± 407.0	4.88 ± 7.13	16.7 ± 544.8	U = 581.50^*^	< 0.001^*^

*SRD* salt-restricted diet, *SUD* salt-unrestricted diet, *BMI* body mass index, *ECW* extracellular water, *ICW* intracellular water, *TBW* total body water, *FMI* fat mass index, *SMI* skeletal muscle index, *BMR* basal metabolic rate, *TR PMM* trunk protein muscle mass, *M* ± *SD* mean ± standard deviation, *IQR* interquartile range, *U* Mann–Whitney U test, *t* Student’s t-test, *P* *p*-value (*statistical significance at p* ≤ *0.05*)

*Statistically significant

### Nutritional risk assessment

A significantly higher proportion of patients in the SUD group were at moderate nutritional risk (75% vs. 25%, *P* = 0.001), whereas the SRD group had a higher prevalence of high nutritional risk (58.1% vs. 41.9%). Although the mean nutritional risk score did not differ significantly between the groups (*P* = 0.139), the distribution of risk categories suggests that salt restriction might influence nutritional status (Table 3).

**Table 3:** Comparison between the two studied groups according to nutritional assessment

	**All (102)**		**SRD (No = 46)**		**SUD (No = 56)**			
	**No**	**%**	**No**	**%**	**No**	**%**		
Moderate risk	40	39.2	10	25.0	30	75.0	χ^2^ = 10.736^*^	0.001^*^
High risk	62	60.8	36	58.1	26	41.9		
Mean ± SD	2.32 ± 1.06		2.50 ± 1.11		2.18 ± 1.01		U = 1076.0	0.139

*IQR* Inter quartile range, *SD* Standard deviation, *U* Mann Whitney test, *χ*^2^ Chi square test, *p* p value for comparison between the two studied groups, *SRD* salt restricted diet, *SUD* salt unrestricted, no number

^*^statistically significant at *p* ≤ 0.05

### Mortality in both groups

Mortality was significantly higher in the SRD group (67.4%) compared to the SUD group (35.7%) (*P* = 0.001) (Table 4).

**Table 4:** Mortality in both groups

**Mortality**	**Total (No = 102)**		**Result of lab**				**χ**^**2**^	**P**
			**SRD** **(No = 46)**		**SUD** **(No = 56)**			
	**No**	**%**	**No**	**%**	**No**	**%**		
Alive	51	50.0	15	32.6	36	64.3	10.137^*^	0.001^*^
Died	51	50.0	31	67.4	20	35.7		

*χ*^2^ Chi square test, *p* p value for comparing between the two studied groups, *SRD* salt restricted diet, *SUD* salt unrestricted diet, No

^*^Statistically significant at p ≤ 0.05

### Logistic regression analysis for nutritional risk

SRD is an independent risk factor for high nutritional risk (*p* = 0.004, OR = 0.129). Other parameters (Child–Pugh score, MELD, SMI, TBW, PMM) were not significant predictors (Table 5).

**Table 5:** Logistic regression analysis for factors affecting higher Nutritional risk

	**Nutritional Assessment**				**Test of sig**	**Univariate analysis**		**Multivariate analysis**	
	**Moderate risk** **(No = 40)**		**High risk** **(No = 62)**			**OR (LL–UL95%C.I)**	**P**	**P**	**OR (LL–UL95%C.I)**
	**No**	**%**	**No**	**%**					
**Child Score**									
**B**	20	50.0	28	45.2	χ^2^ = 0.228 (0.633)	1.214 (0.548–2.693)	0.633	0.464	1.471 (0.524–4.126)
**C**	20	50.0	34	54.8					
**MELD** m ± SD	14.05 ± 6.18		14.0 ± 4.47		U = 1134. (0.466)	0.998 (0.924–1.078)	0.962	0.481	0.966 (0.876–1.064)
**MELD** N m ± SD	118.8 ± 57.49		114.0 ± 42.72		U = 1110. (0.373)	0.998 (0.990–1.006)	0.628	0.950	1.000 (0.990–1.009)
**SMI** m ± SD	40.01 ± 6.98		145.2 ± 519.4		U = 1028.0 (0.146)	1.002 (0.996–1.008)	0.476	0.903	1.024 (0.704–1.489)
**FFM** m ± SD	55.34 ± 10.47		52.18 ± 13.0		U = 1029.5 (0.149)	0.978 (0.946–1.012)	0.200	0.696	0.934 (0.663–1.315)
**TBW** m ± SD	38.89 ± 7.84		36.43 ± 11.91		U = 1063.0 (0.225)	0.977 (0.939–1.017)	0.251	0.561	1.129 (0.750–1.702)
**PMM** m ± SD	51.58 ± 8.87		90.71 ± 180.8		U = 1074.5 (0.377)	1.004 (0.995–1.013)	0.372	0.888	1.011 (0.864–1.184)
**SUD**	30	75.0	26	41.9	χ^2^=10.736^*^ (0.001^*^)	4.154 (1.731–9.970)	0.001^*^	0.004^*^	0.129 (0.032–0.522)
**SRD**	10	25.0	36	58.1					

*SRD* salt-restricted diet, *SUD* salt-unrestricted diet, *MELD* Model for End-Stage Liver Disease, *MELD-Na* MELD with sodium adjustment, *SMI* skeletal muscle index, *FFM* fat-free mass, *TBW* total body water, *PMM* protein muscle mass, *Phys R* physical resistance, *OR* odds ratio, *CI* confidence interval, *LL* lower limit, *UL* upper limit, *χ*^2^ chi-square test, *U* Mann–Whitney U test, *m* ± *SD* mean ± standard deviation, *P* *p*-value (*statistical significance at p* ≤ *0.05*)

^*^Statistically significant

### Logistic regression for factors affecting sarcopenia

SRD is a strong predictor of sarcopenia (*p* < 0.001, OR = 222.12), suggesting a significant impact of salt restriction on muscle loss. MELD-Na score was also significantly associated with sarcopenia (*p* = 0.039), indicating that electrolyte balance disturbances might contribute to muscle depletion (Table 6).

**Table 6:** Logistic regression for the parameters affecting development of sarcopenia

	**Univariate**		^**#**^**Multivariate**	
	**p**	**OR (LL – UL 95%C.I)**	**p**	**OR (LL – UL 95%C.I)**
**Age (years)**	**0.103**	0.966 (0.928–1.007)	0.337	0.969 (0.910–1.033)
**SRD vs SUD**	< 0.001^*^	15.625 (5.513–44.283)	< 0.001^*^	222.12 (19.7–2504.19)
**Child Score (C vs B)**	**0.394**	0.701 (0.310–1.585)	0.413	0.490 (0.088–2.710)
**MELD Score**	**0.831**	1.009 (0.933–1.091)	0.186	1.117 (0.948–1.317)
**MELD Score Na**	**0.859**	1.001 (0.992–1.009)	0.039	1.016 (1.001–1.032)
**Serum sodium**	**0.426**	1.030 (0.958–1.106)	0.197	0.906 (0.781–1.052)
**Serum potassium**	**0.278**	1.350 (0.786–2.319)	0.084	0.467 (0.197–1.106)
**Nutritional Assessment**	**0.083**	2.167 (0.930–5.201)	0.648	0.685 (0.135–3.484)

*OR* Odd`s ratio, *C.I* Confidence interval, *LL* Lower limit, *UL* Upper Limit, *SRD* salt-restricted diet, *SUD* salt-unrestricted diet, *MELD* Model for End-Stage Liver Disease, *MELD-Na* MELD with sodium adjustment

^#^All variables with *p* < 0.05 was included in the multivariate

^*^Statistically significant at *p* ≤ 0.05

### Regression analysis for factors affecting ascites control

Salt restriction significantly improved ascites control (*p* < 0.001, OR = 2.461) in both univariate and multivariate analyses. Serum potassium was inversely related to ascites control (*p* = 0.042 in univariate analysis, though not significant in multivariate analysis) (Table 7).

**Table 7:** Regression analysis for the parameters affecting control of ascites

	**Univariate**		^**#**^**Multivariate**	
	**p**	**B (LL – UL 95%C. I)**	**p**	**B (LL – UL 95%C. I)**
**SRD vs SUD**	< 0.001^*^	2.506 (2.054–2.958)	< 0.001^*^	2.461 (2.011–2.910)
**Child Score (C vs B)**	**0.285**	0.361 (− 0.305 – 1.027)		
**MELD Score**	**0.504**	1.034 (0.938–1.140)		
**MELD Score Na**	**0.786**	0.001 (− 0.006 – 0.008)		
**Serum sodium**	**0.093**	− 0.048 (− 0.105 – 0.008)		
**Serum potassium**	0.042^*^	− 0.417 (− 0.819–− 0.015)	0.074	− 0.250 (− 0.525–0.025)
**Nutritional Assessment**	**0.066**	− 0.631 (− 1.305–0.042)		

*OR* Odd`s ratio, *C.I* Confidence interval, *LL* Lower limit, *UL* Upper Limit, *SRD* salt-restricted diet, *SUD* salt-unrestricted diet, *MELD* Model for End-Stage Liver Disease, *MELD-Na* MELD with sodium adjustment

^#^All variables with p < 0.05 was included in the multivariate

^*^Statistically significant at *p* ≤ 0.05

### Logistic regression analysis for mortality

Sarcopenia was strongly associated with mortality (*p* = 0.006). Patients on a salt-restricted diet had a significantly higher risk of death (*p* < 0.001, OR = 1.65). Other variables (Child–Pugh score, MELD, TBW, PMM) did not reach statistical significance in multivariate analysis (Table 8).

**Table 8:** Logistic regression analysis for mortality regarding different parameters

	**Mortality**				**Test of sig. (p)**	**Univariate analysis**		**Multivariate analysis**	
	**Alive (*n* = 51)**		**Dead (*n* = 51)**			**OR (LL–UL 95%C.I)**	***P* value**	***P* value**	**OR (LL–UL 95%C.I**
	**No**	**%**	**No**	**%**					
**Child Score**									
B	25	49.0	23	45.1	χ^2^ = 0.157 (0.697)	1.171 (0.538–2.549)	0.692	0.550	1.351 (0.504–3.621)
C	26	51.0	28	54.9					
**Nutritional Assessment**									
Moderate risk	24	47.1	16	31.4	χ^2^ = 2.632 (0.105)	1.944 (0.867–4.360)	0.107	0.626	1.269 (0.487–3.311)
High risk	27	52.9	35	68.6					
**MELD** M ± SD	13.51 ± 5.05		14.53 ± 5.3		U = 1129.500 (0.251)	1.040 (0.963–1.123)	0.321	0.564	1.028 (0.935–1.131)
**MELD Na** M ± SD	121.2 ± 52.26		110.5 ± 44.99		U = 1097.500 (0.174)	0.995 (0.987–1.003)	0.264	0.220	0.994 (0.985–1.003)
Not sarcopenic	48	94.1	38	74.5	χ^2^ = 7.413^*^ (0.006^*^)	5.474 (1.454–20.605)	0.012^*^	0.02*	2.684 (0.545–13.230)
Sarcopenic	3	5.9	13	25.5					
**FFM** M ± SD	54.70 ± 11.10		52.14 ± 13.03		U = 1055.00 (0.100)	0.982 (0.951–1.015)	0.287	0.152	0.821 (0.627–1.075)
**TBW** M ± SD	37.85 ± 10.10		36.94 ± 11.02		U = 1087.00 (0.153)	0.992 (0.955–1.029)	0.662	0.131	1.289 (0.928–1.792)
**PMM** M ± SD	84.50 ± 160.7		65.23 ± 118.0		U = 884.500 ^*^ (0.012 ^*^)	0.999 (0.996–1.002)	0.504	0.236	1.004 (0.998–1.010)
**SUD**	36	70.6	20	39.2	χ^2^ = 10.137^*^ (0.001^*^)	3.720 (1.632–8.479)	0.002^*^	< 0.001*	**1.65(1.19–2.121**
**SRD**	15	29.4	31	60.8					

*SD* Standard deviation, *U* Mann Whitney test, *OR* Odd`s ratio, *C.I* Confidence interval, *t* Student t-test, *LL* Lower limit, *UL* Upper Limit, *χ*^2^ Chi square test, *FET* Fisher Exact test, *p* p value for comparing between the studied groups, *BMI* Body Mass Index, *SMI* Skeletal Muscle Index, *PMM* Segmental Muscle Mass, *TR PMM* Trunk muscle mass

^*^Statistically significant

## Discussion

This prospective observational cohort study provides critical insights into the complex interplay between salt restriction, nutritional status, sarcopenia, and mortality in cirrhotic patients with grade III ascites. Our findings challenge the conventional paradigm that strict dietary sodium restriction is universally beneficial for ascites management. Instead, our results suggest that while sodium restriction may theoretically reduce fluid retention, it is associated with significant adverse effects, including higher mortality rates, increased prevalence of sarcopenia, and poorer nutritional status. These findings align with emerging literature questioning the indiscriminate application of sodium restriction in cirrhotic patients and highlight the need for a more nuanced, individualized approach to dietary management [18].

Malnutrition is a well-recognized complication in patients with cirrhosis, with prevalence rates reaching up to 80% [5]. Protein-energy malnutrition, compounded by increased metabolic demands and anorexia, is exacerbated by excessive sodium restriction, as demonstrated in our study. One of the most striking findings was the significant increase in nutritional risk among patients on a salt-restricted diet. Patients in the SRD group had a higher proportion of high nutritional risk (58.1%) than those in the SUD group (41.9%) (*p* = 0.001). The logistic regression analysis revealed that SRD was an independent predictor of high nutritional risk (OR = 0.129, *p* = 0.004). This suggests that excessive sodium restriction may contribute to insufficient caloric and protein intake, leading to muscle wasting. Similar findings were reported by Sorrentino et al., emphasizing that restricting sodium without compensatory nutritional strategies may lead to deterioration in muscle and protein stores [19].

Few studies, with varying methodological quality and inconsistent findings, have examined the effects of salt restriction on nutrition in patients with cirrhosis. Soulsby et al. found that patients without salt restrictions experienced reduced ascites, better nutritional status, and shorter hospital stays [20]. Similarly, Gu et al., in the largest study to date, randomized 200 patients with ascites into unrestricted (8.8 g NaCl) and restricted (4.2 g NaCl) sodium groups, finding that the unrestricted group experienced better renal function, higher calorie intake, increased serum albumin, and shorter hospital stays, along with greater ascites resolution (*P* = 0.001) [21]. Similarly, Sorrentino et al. observed that the sodium-restricted group required paracentesis more frequently and had higher mortality rates [19]. Morando et al. in a study of 120 outpatients, noted that in the salt-restricted diet group (4.6 g NaCl/day) there was reduced caloric intake by 20%, with only 30.8% adhering to the diet. Many patients misunderstand their sodium intake levels, likely due to inadequate dietary guidance, leading some to unintentionally lower their calorie intake to reduce salt intake [22].

The present study highlights the critical role of sarcopenia in cirrhotic patient outcomes. Sarcopenia was significantly more prevalent in the SRD group (*p* < 0.006), with a lower skeletal muscle index (SMI) and trunk protein muscle mass (TR PMM). More importantly, sarcopenia was an independent predictor of mortality (OR = 2.684, *p* = 0.02) in our multivariate analysis. These findings corroborate previous reports by Montano-Loza et al. and Kalafateli et al., who identified sarcopenia as an independent risk factor for poor prognosis in cirrhotic patients [23, 24].

The lower PMM in the SRD group may stem from dietary restrictions and cirrhosis-related catabolism, whereas fluid overload in the SUD group may artificially inflate PMM readings because increased extracellular fluid can distort body composition. It is likely that the results of a small study by Soulsby et al., who reported reduced dry body weight and mid-arm muscle circumference over four weeks on a low-sodium diet, although this timeframe is too brief to assess long-term impacts [19]. The pathophysiological mechanisms underlying this association likely involve increased catabolism due to inadequate protein intake, loss of muscle mass secondary to sodium restriction-induced metabolic alterations, and impaired nitrogen balance [25].

The primary rationale for sodium restriction in cirrhosis is to reduce fluid retention and decrease the need for paracentesis [26]. This study data suggests that sodium restriction was necessarily translated into superior ascites control.

Remarkedly, the SUD group showed significantly higher modified weight than in the SRD group (*p* = 0.001). Total body water (TBW) was also significantly higher in the SUD group (mean 40.31 ± 12.08) than in the SRD group (mean 33.84 ± 6.86, *p* < 0.001), which could indicate that patients on an unrestricted diet retained more fluid, leading to higher TBW. Water retention increases body weight without necessarily reflecting improved nutritional or muscle status, as it primarily represents extracellular fluid accumulation [27, 28].

Hence, these results demonstrate that salt restriction significantly improves ascites control, and the regression analysis confirmed that SRD was an independent predictor of improved ascites control (OR = 2.461, *p* < 0.001). These findings align with previous studies supporting sodium restriction as a fundamental component of ascites management. However, our study suggests that while ascites control is enhanced, it comes at the cost of worsening nutritional status, warranting careful dietary adjustments.

Contradictory, was the observation reported by Ruiz-Margáin et al., who noted that a less restrictive sodium approach did not worsen ascites control but improved nutritional outcomes in cirrhotic patients [29].

While paracentesis frequency is a widely used marker for ascites control, it does not fully capture the complex interplay of fluid balance, nutritional status, and renal function in cirrhotic patients. To address this limitation, our study also considered changes in body weight, TBW measured by BIA, and serum sodium levels as additional markers of ascites management. Our results indicate that patients in the SRD group exhibited lower TBW and required fewer paracenteses**,** suggesting better ascites control. However, the significant weight loss observed in the SRD group raises concerns about nutritional depletion and potential muscle loss due to excessive sodium restriction**.** These findings mirror similar results in literature. For instance, Gu et al. and Sorrentino et al. reported that patients following a less restrictive sodium regimen had better nutritional outcomes, including higher caloric and protein intake, which contributed to improved ascites control and reduced need for paracentesis [20, 21]. Bernardi et al. also highlighted that excessive sodium restriction can exacerbate malnutrition and muscle wasting, ultimately leading to more frequent ascites’ recurrence [24]. These findings highlight the delicate balance between sodium restriction, fluid management, and nutritional status in cirrhotic patients. Future studies should integrate longitudinal assessments of ascitic fluid volume, renal function, and body composition to provide a more comprehensive evaluation of ascites control in response to dietary interventions.

One of the most striking findings of our study is the significantly higher mortality rate in the SRD group (67.4%) compared to the SUD group (35.7%, *p* = 0.001). This association persisted after adjusting for confounders, with logistic regression confirming salt restriction as an independent predictor of mortality (OR = 3.72, 95% CI: 1.63–8.48, *p* = 0.002). This raises important concerns regarding the conventional wisdom advocating for stringent sodium restriction in cirrhotic patients with ascites. Previous studies have reported conflicting results regarding the impact of sodium intake on mortality. Pashayee-Khamene et al. found that moderate sodium restriction, rather than severe limitation, was associated with improved survival in cirrhotic patients [30]. In contrast, Sorrentino et al. demonstrated that patients on a strict salt-restricted diet without nutritional support had a 3.9-fold higher risk of mortality within one year [21]. Our findings align with these studies, reinforcing the notion that excessive sodium restriction may have unintended deleterious effects on patient survival.

A balanced dietary approach is essential for cirrhotic patients to mitigate the nutritional risks of salt restriction, including muscle loss and malnutrition. Adequate protein intake (1.2–1.5 g/kg/day), high-quality protein sources, and BCAA supplementation help preserve muscle mass, while frequent meals and bedtime snacks counteract catabolism. Moderate sodium restriction (5–7 g salt/day) optimizes ascites control without causing excessive volume contraction or hyponatremia. Additionally, potassium-rich foods and calcium/vitamin D supplementation support electrolyte balance and bone health. Future research should explore structured nutritional interventions to enhance both ascites management and overall nutritional outcomes.

This study has several strengths, including its prospective design, which allows for a more accurate assessment of dietary sodium adherence and its effects on clinical outcomes compared to retrospective analyses. The comprehensive evaluation of multiple clinically relevant endpoints, such as sarcopenia, nutritional status, and mortality, provides a holistic understanding of sodium restriction's impact on cirrhotic patients. Additionally, the use of validated methodologies, including the Royal Free Hospital-Nutritional Prioritizing Tool (RFH-NPT) for nutritional assessment and bioelectrical impedance analysis for sarcopenia evaluation, strengthens the study’s reliability. The sample size was adequate for detecting significant differences in mortality and sarcopenia, as confirmed by post hoc power analysis, ensuring robustness in the findings.

However, there are limitations to consider. Being a single-center study, the generalizability of the findings to broader populations may be limited. Furthermore, while baseline sarcopenia was assessed, the absence of serial sarcopenia measurements precludes the evaluation of its progression over time. Lastly, the six-month follow-up period, though adequate for short-term mortality and nutritional impact evaluation, does not account for long-term outcomes related to sodium intake, necessitating further longitudinal studies.

Our study underscores the need for an individualized, patient-centered approach to dietary sodium management in cirrhotic patients. Rather than a blanket recommendation for strict sodium restriction, a tailored approach that considers each patient’s nutritional status, sarcopenia risk, and fluid balance is warranted. Future multicenter, randomized controlled trials are necessary to refine sodium intake recommendations and develop evidence-based dietary guidelines that optimize both ascites control and nutritional status.

## Conclusion

Our findings challenge the long-standing clinical dogma that strict salt restriction is universally beneficial for cirrhotic patients with ascites. The results indicate that severe sodium restriction despite being beneficial to ascites control- may worsen nutritional status, increase sarcopenia prevalence, and lead to higher mortality rates. These findings emphasize the need for a more individualized approach to dietary sodium management, balancing ascites control with the preservation of nutritional and functional status.

## Data Availability

“Data used to support the findings of this study are available from the corresponding author upon request.”

## Abbreviations

DAAS: Direct-acting antiviral
DSRQ: Dietary Sodium Restriction Questionnaire
FMI: Fat mass index
INR: International normalized ratio
RFH-NPT: Royal free hospital-nutritional prioritizing tool
SMI: Skeletal muscle index
SRD: Salt Restriction diet
SUD: Salt Un-Restricted diet
